# High-frequency ultrasound in patients with seronegative rheumatoid arthritis

**DOI:** 10.1038/s41598-022-25958-w

**Published:** 2022-12-09

**Authors:** Junkui Wang, Miao Wang, Qinghua Qi, Zhibin Wu, Jianguo Wen

**Affiliations:** 1 Ultrasound Department, First Affiliated Hospital of Zhengzhou University, Zhengzhou, 450052 China; 2 Surgery Department, First Affiliated Hospital of Zhengzhou University, Zhengzhou, 450052 China; 3 Henan Joint International Pediatric Urodynamic Center, The First Affiliated Hospital of Zhengzhou, Zhengzhou, 450052 China

**Keywords:** Osteoarthritis, Rheumatoid arthritis

## Abstract

This study aimed to investigate the value of high-frequency ultrasound (HFUS) in differentiation of the seronegative rheumatoid arthritis (SNRA) and osteoarthritis (OA) and in the diagnosis of SNRA. 83 patients diagnosed with SNRA (SNRA group) and 40 diagnosed with OA (OA group) who received HFUS were retrospectively analyzed. The grayscale (GS) scores, power Doppler (PD) scores, and bone erosion (BE)scores were recorded, and added up to calculate the total scores of US variables. The correlations of the total scores of US variables with the 28-joint disease activity score (DAS28), erythrocyte sedimentation rate (ESR) and C-reactive protein (CRP) were analyzed. The diagnostic efficacy of the total scores of US variables for SNRA was assessed. In the SNRA group, the detection rate of abnormal US findings in the joints and tendons by GS and PD as well as BE was higher than those in the OA group. There were significant differences between the two groups in GS scores and PD scores of joints and tendons, and BE scores of joints (*P* < 0.05). In the SNRA group, the total scores of most US variables were positively correlated with CRP, ESR, and DAS28 (*P* < 0.05), while such correlations were not observed in the OA group (*P* > 0.05). Among different US variables, the diagnostic value of total PD scores of the joints was the highest for SNRA. HFUS could be used to differentiate SNRA from OA and make a diagnosis of SNRA based on joint and tendon synovial sheath assessment.

## Introduction

Rheumatoid arthritis (RA) is a chronic disease with synovitis as a primary pathological feature. This disease may lead to cartilage and bone erosion (BE) and injury, joint deformity, or even long-term disability^1^. Autoantibodies, such as rheumatoid factor (RF) and anti-cyclic peptide containing citrulline antibody (ACPA) are considered to be of diagnostic value for RA. However, the autoantibodies above may be absent in the serum of some RA patients. Pratt et al*.*^2^ defined RA negative for serum RF and ACPA as seronegative RA (SNRA), a disorder most common in those aged above 50. Since SNRA has both the invasiveness of RA and the serologically occult nature of osteoarthritis (OA), it is difficult to make a diagnosis of SNRA at an early stage. It is often necessary to differentiate SNRA from OA. Aside from clinical symptoms and laboratory tests, radiological imaging is also important.

High-frequency ultrasound (HFUS) has become one of the major imaging tools for clinical diagnosis alongside X-ray, Computed Tomography (CT), and magnetic resonance imaging (MRI) for the musculoskeletal system. So far, HFUS has been used in the daily clinical practice in rheumatology as a powerful tool^3–5^. HFUS is generally known for its higher soft tissue resolution, which also detects erosive lesions of cartilages and bones and blood flow signals in the thickened synovium. Besides, this imaging method is featured by time-saving, non-invasiveness, repeatability, and dynamic observation, and is therefore potentially applied to the diagnosis of joint lesions in RA patients^6–8^. The sensitivity and specificity of HFUS in detecting synovitis of RA patients are similar to MRI^6,9^. Moreover, the intra-rater and inter-rater reliability of HFUS is high^7,10^, the use of which in joint examination has been recommended in the new ACR/EULAR classification criteria^11^. Grayscale (GS) and power Doppler (PD) ultrasonography provides useful information for staging and diagnosis of RA^8,12,13^. In the present study, the GS and PD ultrasound features of SNRA and OA were analyzed, and the purpose was to facilitate early diagnosis of SNRA.

## Materials and methods

### Patients and eligibility criteria

We retrospectively analyzed 140 patients suspected of RA or OA at our department from January 2017 to March 2021. All patients had swollen and tender wrist or proximal interphalangeal joints. After the 6-month follow-up, 123 eligible patients (5 cases with psoriatic arthritis, 4 cases with spondyloarthropathy, 4 cases with gout, 2 cases with systemic lupus erythematosus and 2 cases not followed up) were divided into the SNRA group (n = 83) and OA group (n = 40) depending on clinical diagnosis. Informed consent was obtained from all participants. The protocol was approved by the medical ethics committee of Zhengzhou University.

The diagnosis of RA and OA was made according to the 2010 American College of Rheumatology (ACR)/European League Against Rheumatism (EULAR) classification criteria and the 1990 ACR diagnostic criteria or EULAR recommendations, respectively^14–16^.

 Inclusion criteria: (1) RA patients were negative serological results for ACPA and RF; and all OA patients met the diagnosis criteria including osteophytes on radiograph and clinical examinations. (2) HFUS of the metacarpophalangeal joints (MCP) and proximal interphalangeal joints (PIP) and wrist joints upon the initial visit, with bilateral hand X-ray (postero-anterior view) and other intact data.

Exclusion criteria: (1) For SNRA patients, other articular diseases, such as systemic lupus erythematosus, gout, psoriatic arthritis, spondyloarthropathy, reactive arthritis, viral arthritis, infection and trauma, etc.; For OA patients, trauma or operation of the hands within 6 months, or an intra-articular injection within 3 months prior to inclusion, carpal tunnel syndrome or another inflammatory joint disease (e.g.,, crystal arthropathy, such as gout or chondrocalcinosis with clinical symptoms, RA, psoriatic arthritis, etc.) (2) mental disorders; (3) history of joint replacement; absence or deformity of finger and wrist joints; (4) severe injury of the hemopoietic, cerebral, cardiac, and renal systems or severe malnutrition.

The clinical synovitis indicators and the general information of patients were collected: age, gender, drinking, smoking, course of disease; swollen joint count (SJC) and tender joint count (TJC), morning stiffness duration, general health (GH), erythrocyte sedimentation rate (ESR), ACPA, 28 activity scores in 28 joints (DAS28), C-reactive protein (CRP), and RF. DAS28 was calculated as follows: DAS28 = 0.56 × sqr(TJC) + 0.28 × sqr(SJC) + 0.70 × LnESR + 0.014 × GH^17^.

### US examination

The Samsung RS85 Ultrasound System (Samsung Medison Co, Ltd.) and an L3-12A ultrasound transducer (frequency 3–12 MHz) were used. PD examination was performed at a pulse repetition frequency of 0.40 kHz and a gain of 54. All examinations were performed by one ultrasound physician who was blinded to patients' laboratory and clinical indicators and well trained in musculoskeletal ultrasound. Ultrasonography was performed on 22 joints at the dorsal and palmar sides of the two wrists for each patient, including bilateral MCP of digits 1–5 and bilateral PIP. Meanwhile, for each patient, ultrasonography was performed on 24 tendons on the dorsal and palmar sides of the two hands, including flexor carpi radialis, flexor tendons of the five digits, and extensor tendon compartments I-VI of the wrist. The lesion interpretation, scanning plane, and examination procedures of joints and tendon synovial sheaths were determined based on the Scoring systems proposed by Outcome Measure in Rheumatology in Clinical Trails (OMERACT)^18–21^. The joint GS, PD, and BE grading was performed according to the semi-quantitative scoring system using a 0–3 scale proposed by Szkudlarek et al.^22^. (GS: 0, no synovial thickening (ST); 1, ST without bulging over the line linking tops of the periarticular bones; 2, ST and bulging over the line linking tops of the periarticular bones; 3, ST and bulging over the line linking tops of the periarticular bones and with extension to at least one of the bone diaphysis. PD: 0, absence or minimal flow; 1, single vessel signal; 2, confluent vessel signals in < 50% of the joint area; 3, confluent vessel signals in ≥ 50% of the joint area. BE: 0, regular bone surface; 1, irregular bone surface without a defect visualised in 2 planes; 2, a defect in the surface of the bone visualised in 2 planes; 3, bone defect leading to extensive bone destruction). The highest value observed for each joint at any level would prevail. Measurement was performed at the maximum cross-section of each tendon synovial sheath. The GS and PD scores for tenosynovitis were semi-quantitated using a 0–3 scale^20,21^. (GS:0 = normal; 1 = minimal; 2 = moderate; 3 = severe. PD: 0 = no peritendinous Doppler activity (PDA); 1 = focal PDA; 2 = multifocal PDA; 3 = diffuse PDA). The following US variables were determined: GS scores of the joint, PD scores of the joint, BE scores of the joint, GS scores of the tendon synovial sheath, and PD scores of the tendon synovial sheath. The GS, PD and BE scores of each joint were added up as the total GS, PD and BE scores (0–66 points) of joints for each patient, respectively. The GS and PD scores of each tendon synovial sheath were added up as the total GS and PD scores of tendon synovial sheaths (0–72 points), respectively.

### Statistical analysis

GraphPad Prism 5.04 (GraphPad Software Inc., 2012, La Jolla, CA) and SPSS 20.0 (SPSS Inc, Chicago, IL, USA) were used for plotting and statistical analyses, respectively. Counts were described as percentages or cases. Measurements which obeyed a normal distribution were described as ${\overline{\text{x}}}\pm {\text{s}}$. The total scores of US variables were described as ${\overline{\text{x}}}\pm {\text{s}}$ Mann–Whitney U test or t-test was used to analyze the inter-group differences in measurements. Fisher's exact test or χ^2^ test was used to analyze categorical variables in the two groups. Mann–Whitney U test was used to analyze categorical variables. Pearson's correlation coefficient was employed to analyze the potential correlations between continuous variables. The receiver operating characteristic (ROC) curve was plotted to assess the predictive value of US variables for SNRA. The score corresponding to the maximal Youden index (sensitivity + specificity − 1) was used as the cut-off value. The specificity and sensitivity were calculated from the ROC curve. *P* < 0.05 indicated a significant difference.

### Ethics approval

The study was approved by the medical ethics committee of Zhengzhou University (Henan, China) and was in accordance with the principles of the 1964 Declaration of Helsinki and its later amendments or comparable ethical standards.

### Consent to participate

The legal guardian of all participants were informed of the purpose of the study. Written informed consent was obtained from them.

## Results

### Demographic characteristics

The clinical data of patients from the SNRA group and the OA group are shown in Table 1. The two groups of patients were not significantly different in gender, BMI, smoking status, alcohol use, and Disease duration (P > 0.05). The CRP, ESR, and DAS28 levels of the SNRA group were significantly higher than those of the OA group (*P* < 0.05).

**Table 1:** Demographic, laboratory and clinical features.

Variables	SNRA(n = 83)	OA (n = 40)	*P* value
Age, years^a^	57.07 ± 9.26(35–93)	59.23 ± 10.07(45–81)	0.241
Gender (female/male)	54 /29	30/10	0.267
BMI(kg/m^2^)^a^	22.35 ± 3.19 (10–37)	22.44 ± 3.55(11–36)	0.888
Smoking( Yes/ No)	25/58	8/32	0.235
Drinking( Yes/ No)	20/63	8/32	0.612
Disease duration (weeks)^a^	38.90 ± 22.67 (2–288)	40.70 ± 25.72 (3–320)	0.707
CRP (mg/L)^a^	37.50 ± 45.76(0–192)	2.05 ± 1.29(0–5.75)	0.000
ESR (mm/h)^a^	38.97 ± 33.27(3–108)	9.33 ± 6.58(1–26)	0.000
DAS28^a^	5.56 ± 1.42(2.6–8.6)	2.47 ± 0.91(0.9–4.4)	0.000

Note: ^a^Mean ± SD (range).

Abbreviations: SNRA, seronegative rheumatoid arthritis; OA, osteoarthritis; BMI, Body Mass Index; CRP, C-reactive protein; ESR, erythrocyte sedimentation rate; DAS28, disease activity scores in 28 joints.

### US variables in SNRA and OA patients

**Figure 1:**
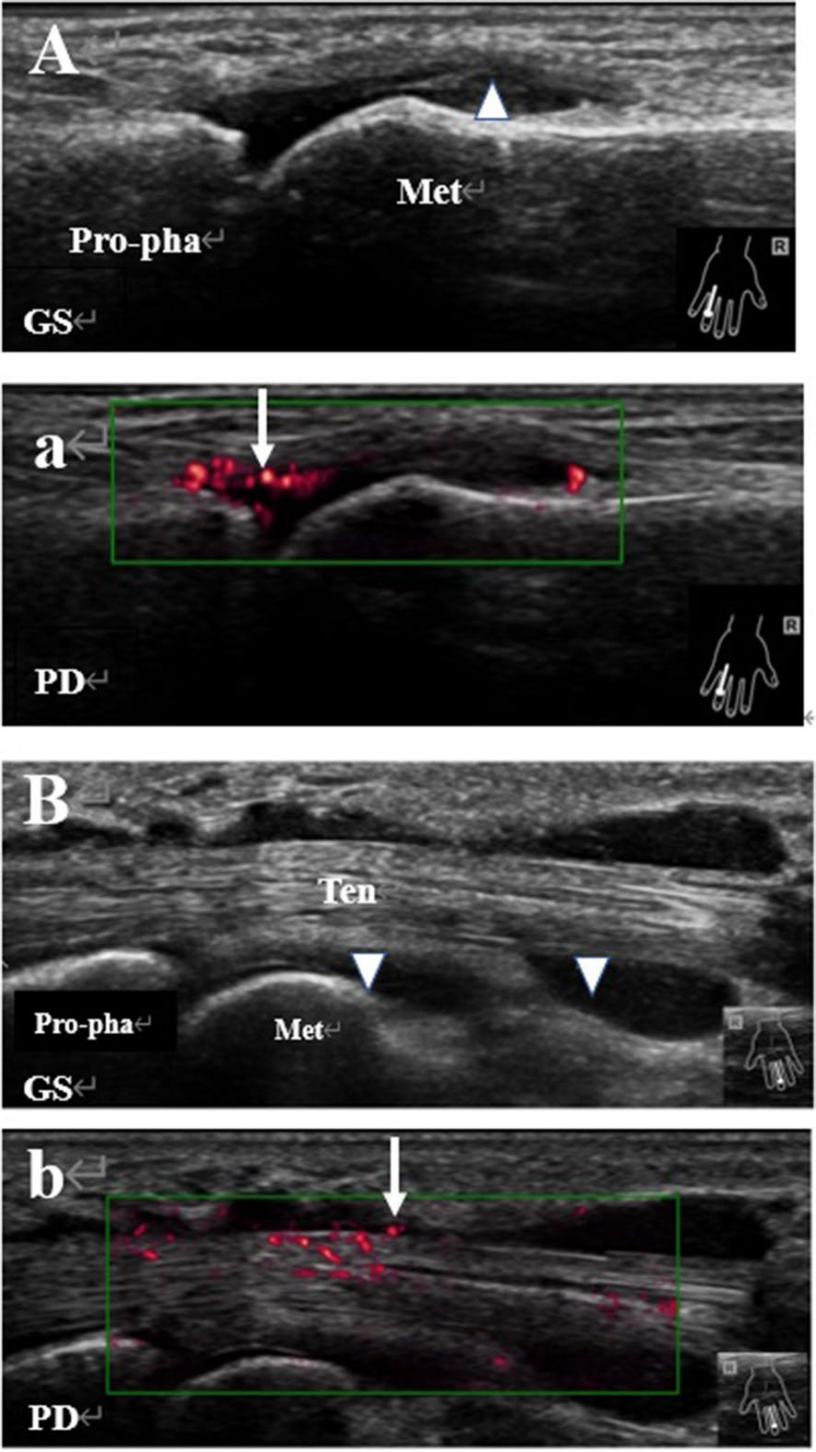
Representative images of synovitis and tenosynovitis in a patient with SNRA. (**A**) Synovitis in the fourth metacarpophalangeal joint of the right hand, with the GS grade of 3 (triangle). a. Blood flow signals in the thickened synovium upon PD (arrow), with the PD grade of 2; (**B**) Tenosynovitis in the flexor tendon of the third digit of the right hand, with the GS grade of tendon synovial sheath of 3 (triangle); b. Blood flow signals observed near the tendon synovial sheath upon PD (arrow), with the PD grade of 2. Abbreviations: GS Grayscale, PD power Doppler, Met metacarpal, Pro-pha proximal phalanx, Ten flexor tendon.

In the SNRA group, 1,826 joints were found abnormal, including 1,494 (81.82%) by GS and 934 (51.15%) by PD. There were 690 joints (37.79%) with BE. The detection rate of abnormal US findings were all higher than those in the OA group, which were 492 (55.91%) (χ^2^ = 15.802, *P* = 0.000), 25 (2.84%) (χ^2^ = 58.447, *P* = 0.000) and 12 (1.35%) (χ^2^ = 43.606, *P* = 0.000), respectively. In the SNRA group, 1,992 tendons were found abnormal, including 778 by GS (39.06%) and 615 by PD (30.97%). They were also significantly higher than those in the OA group, which were GS 74 (7.71%) (χ^2^ = 26.728, *P* = 0.000) and PD 9(0.94%) (χ^2^ = 33.282, *P* = 0.000), respectively. The typical ultrasound images of an SNRA patient with arthrosynovitis and tenosynovitis are shown in Fig. 1.

The average total GS score of joints in the SNRA group was 39.87 ± 11.41, and the average total PD score of joints was 18.18 ± 12.64. The total average BE score was 9.17 ± 6.70, and the average total GS score of tendon synovial sheaths was 17.96 ± 14.76. The average total PD score of tendon synovial sheaths was 14.42 ± 13.96. These scores were all higher than those in the OA group, which were 21.78 ± 6.62, 0.95 ± 1.83, 0.43 ± 1.11, 2.00 ± 2.83, and 0.23 ± 0.58, respectively.

**Figure 2:**
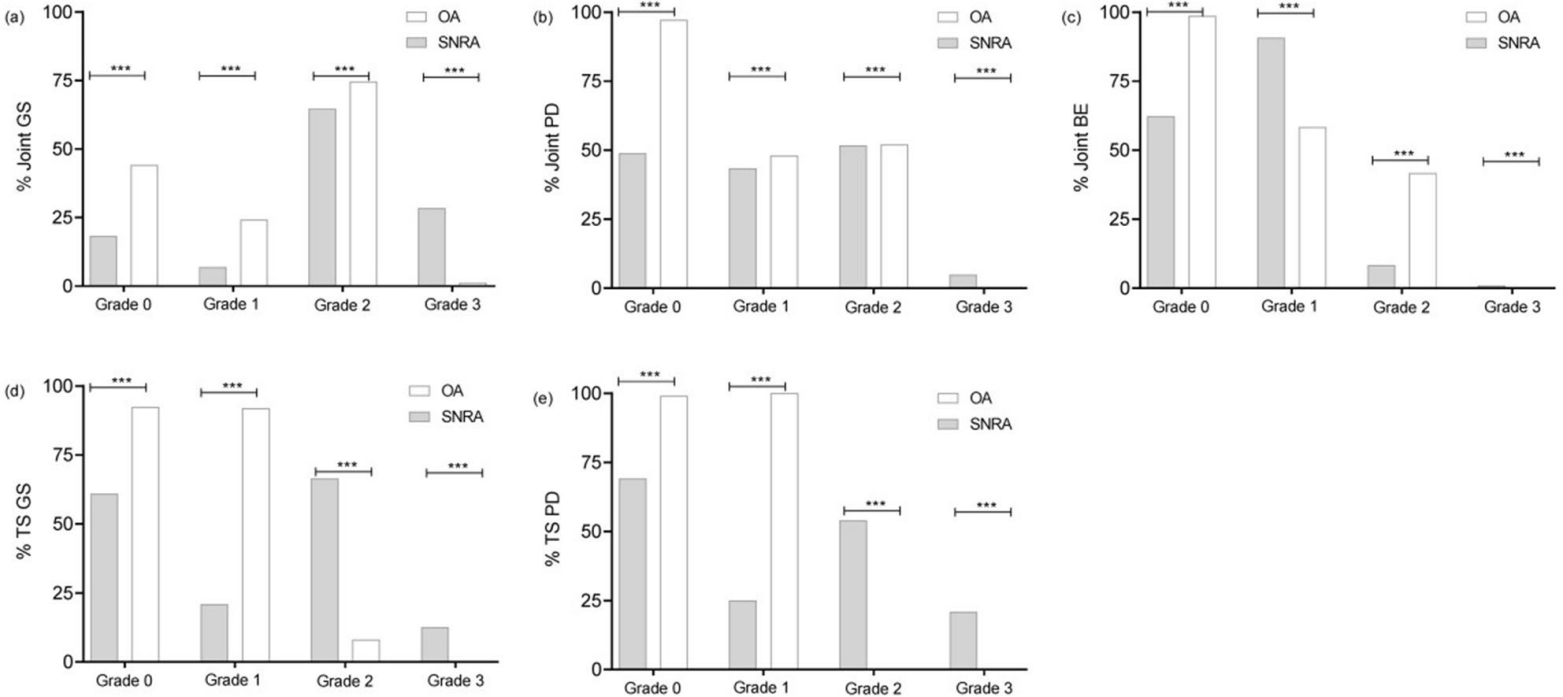
Distribution of the grade of US variables in SNRA and OA patients. Note: ***P < 0.001. Abbreviations: SNRA, seronegative rheumatoid arthritis; OA, osteoarthritis; GS, grayscale; PD, power Doppler; BE, bone erosion; TS, tenosynovitis.

The average GS score of joints was 1.81 ± 0.52 in the SNRA group compared to 0.99 ± 0.30 in the OA group, with the predominance of GS grade 2 in abnormality joint GS in both groups. The average PD score of joints was 0.83 ± 0.57 in the SNRA group compared to 0.04 ± 0.08 in the OA group, with the predominance of PD grade 2 in abnormality joint PD in both groups. The average BE score was 0.42 ± 0.31 in the SNRA group compared to 0.02 ± 0.05 in the OA group, with the predominance of grade 1 in abnormality joint BE in both groups. The average GS score of tendon synovial sheaths was 0.75 ± 0.62 in the SNRA group compared to 0.08 ± 0.12 in the OA group. GS grade 2 was more predominant in abnormality tenosynovitis GS in the SNRA group, and GS grade 1 in abnormality tenosynovitis GS was more dominant in the OA group. The average PD score of tendon synovial sheaths was 0.60 ± 0.58 in the SNRA group compared to 0.01 ± 0.024 in the OA group. PD grade 2 in abnormality tenosynovitis PD was more dominant in the SNRA group, and PD grade 1 in abnormality tenosynovitis PD was only observed in the OA group. The two groups were significantly different in the distribution of GS scores of joints, PD scores of joints, BE scores of joints, GS scores of tendon synovial sheaths, and PD scores of tendon synovial sheaths (Table 2, Fig. 2).

**Table 2:** Comparison of the grade of US variables in SNRA and OA patients (n).

	Joint GS		Joint PD		Joint BE		TS GS		TS PD	
	SNRA	OA	SNRA	OA	SNRA	OA	SNRA	OA	SNRA	OA
Grade 0	332	388	892	855	1136	868	1214	886	1377	951
Grade I	103	119	405	12	626	7	163	68	154	9
Grade II	967	367	483	13	57	5	517	6	332	0
Grade III	424	6	46	0	7	0	98	0	129	0
Z value	−19.669		−24.059		−20.074		−18.553		−18.649	
*P* value	0.000		0.000		0.000		0.000		0.000	

Abbreviations: SNRA, seronegative rheumatoid arthritis; OA, osteoarthritis; GS, grayscale; PD, power Doppler; BE, bone erosion; TS, tenosynovitis.

### The correlation between the total scores of US variables and CRP, ESR, DAS28

Except that CRP in the SNRA group was not significantly correlated with total BE scores, total GS scores of joints, total PD scores of joints, total BE scores, total GS scores of tendon synovial sheaths, and total PD scores of tendon synovial sheaths were positively correlated with ESR, CRP and DAS28 (*P* < 0.05). In the OA group, the total scores of US variables were not correlated with CRP, ESR, or DAS28 (*P* > 0.05) (Table 3).

**Table 3:** Correlation between total scores of US variables and CRP, ESR, and DAS28 in SNRA group and OA group.

		Joint GS		Joint PD		Joint BE		TS GS		TS PD	
		r	p	r	p	r	p	r	p	r	p
SNRA group	CRP	0.235	0.032	0.223	0.043	0.168	0.128	0.347	0.001	0.379	0.000
	ESR	0.295	0.007	0.282	0.010	0.200	0.007	0.379	0.000	0.372	0.001
	DAS28	0.810	0.000	0.792	0.000	0.602	0.000	0.659	0.000	0.611	0.000
OA group	CRP	0.093	0.568	0.263	0.102	0.309	0.052	0.046	0.776	0.012	0.940
	ESR	0.285	0.075	0.162	0.319	0.023	0.889	0.283	0.077	0.291	0.068
	DAS28	0.309	0.052	0.136	0.404	0.002	0.992	0.301	0.059	0.218	0.176

Abbreviations: SNRA, seronegative rheumatoid arthritis; OA, osteoarthritis; CRP, C-reactive protein; ESR, erythrocyte sedimentation rate; DAS28, disease activity scores in 28 joints; GS, grayscale; PD, power Doppler; BE, bone erosion; TS, tenosynovitis.

### Sensitivity and specificity of total scores of US variables for SNRA

The diagnostic performance of total scores of diverse US variables for SNRA is shown in Table 4. The diagnostic value of all the five US variables, namely, total GS scores of joints, total PD scores of joints, total BE scores, total GS scores of tendon synovial sheaths, and total PD scores of tendon synovial sheaths, was high (AUC > 0.85). Among them, the diagnostic value of total PD scores of joints was the highest for SNRA. The cut-off value of total PD scores of joints was 4, and the corresponding AUC was 0.97. When the total PD score of joints was 4, the sensitivity of the total PD score of joints for the diagnosis of SNRA was 90.36%, the specificity was 92.50%, the positive predictive value was 70.09%, and the negative predictive value was 82.22%.

**Table 4:** Sensitivity and specificity of ultrasound variables for SNRA.

Variables	CP (points)	Sensitivity (%)	Specificity (%)	PPV (%)	NPV (%)	AUC
Joint GS	≥ 25	95.18	72.50	68.70	87.88	0.92
Joint PD	≥ 4	90.36	92.50	70.09	82.22	0.97
Joint BE	≥ 3	81.93	95.00	73.12	71.70	0.94
TS GS	≥ 7	71.08	92.50	78.67	60.65	0.87
TS PD	≥ 3	77.11	87.50	75.29	64.82	0.88

Abbreviations: SNRA, seronegative rheumatoid arthritis; GS, Grayscale; PD, power Doppler; BE, bone erosion; TS, tenosynovitis; CP, cut-off point; PPV, positive predictive value; NPV, negative predictive value; AUC, area under the receiver operating characteristic curve.

## Discussion

The US variables of joints and tendon synovial sheaths were compared between the SNRA group and the OA group. It was found that the detection rate of abnormal US variables of joints and tendon synovial sheaths and the total scores of US variables in the SNRA group were higher than those in the OA group. The two groups were significantly different in the distribution of the grade of US variables. The total scores of most US variables in the SNRA group were positively correlated with CRP, ESR and DAS28. However, no such correlations were observed in the OA group. Moreover, the total scores of US variables had a high diagnostic value for SNRA. Among the variables, the total PD score of joints had the highest diagnostic value for SNRA, and the corresponding the cut-off value was 4. HFUS was used to assess joints and tendon synovial sheaths, which facilitated the differential diagnosis of SNRA and OA and the clinical diagnosis of SNRA.

RA is a chronic, autoimmune disease primarily involving the joints, which is featured by persistent synovitis and may cause cartilage and bone injury and deformity of multiple joints^1^. Early diagnosis and treatment are critical for RA. Several specific autoantibodies are usually available in the serum of RA patients. Among them, RF and ACPA are indicative factors for the diagnosis of RA^23^. At present, SNRA refers to RA with negative serological results for RF and ACPA although the patients conform to the 2010 ACR/EULAR diagnostic criteria^2^. SNRA accounts for 10–30% of all RA patients. However, there are controversies about SNRA, and it may be wrong to consider SNRA as a mild type of RA^24^. Both SNRA and OA are more common in the middle-aged and elderly. SNRA is featured by invasiveness of RA and the serologically occult nature of OA. It is sometimes difficult to differentiate SNRA from OA based on clinical manifestations alone. SNRA is very likely to be misdiagnosed as OA. Usually, a long observation window is required before confirmed diagnosis of SNRA. For this reason, it is difficult to identify and manage SNRA at an early stage^1,25,26^. Radiological imaging is also significant for the diagnosis of SNRA apart from clinical symptoms and laboratory tests.

With a high-frequency probe, the diagnostic value of US in synovitis, tenosynovitis, and BE is not less than MRI^4,9^. US is also known for its high intra-rater and inter-rater reliability^7,10^. Given all these features, US is a good choice for early diagnosis of SNRA, which differentiates SNRA from OA. GS ultrasound allows direct visualizing of the morphology and quantity (hypertrophy) of the synovial tissue. PD ultrasound can be used to visualize blood flow in joints and tendon synovial sheaths, with a high sensitivity and specificity for inflammation^8,12^. In the present study, the detection rate of abnormal joints upon GS and PD and the total GS score of joints and the total PD score of joints in the SNRA group were also higher than those of the OA group, respectively. Although the number of joints with GS grade 2 and PD grade 2 was larger in both groups, the average GS score of joints and the average PD score of joints in the SNRA group were higher than those of the OA group. Besides, the two groups were significantly different in the distribution of the GS and PD grade. The diagnostic value of total GS scores of joints and total PD scores of joints was higher for SNRA (AUC > 0.85). The diagnostic value of total PD scores of joints was the highest, and the corresponding the cut-off value was 4. Thus, blood flow signals detected by PD in at least two involved joints with a cumulative score of 4 indicated SNRA. GS and PD ultrasound of joints in the two groups was helpful for differential diagnosis of SNRA and OA.

 BE is a primary feature of RA, which has been widely recognized for its role in the pathogenesis, disease, and prognosis of RA^27^. BE is a joint injury in RA, and closely related to irreversible joint function loss^28^. Early diagnosis of RA is important for disease management and prevention of further joint injury^29,30^. HFUS usually reveals locally unsmooth cortex of the articular surface, with disrupted continuity and eroded edge, or even extensive cystic deformation on the bone surface, which may evolve into a bone cyst^19^. HFUS and MRI were similar in detecting early, small erosions on the cortex surface^9,31^. Kosta et al.^32^ reported BE upon MRI in 96.2% of the RA patients with a course shorter than 3 months. Ji et al.^33^ found BE by ultrasound in 44.8% of the RA patients with a course shorter than 2 years. Besides, the number of joints with BE was much larger in RA patients than in non-RA patients. Compared with the OA group, the detection rate of BE, the total BE score, grade of BE, and the average score of BE were higher in the SNRA. The diagnostic value of total BE scores was also high for SNRA (AUC > 0.85). Among the total scores of five US variables, the specificity of the total BE score was the highest, which was 95.00%, but the sensitivity was poor. Severe BE was already indicated upon the initial visit in some SNRA patients (about 9.28%). In fact, these patients were not diagnosed at an early stage. However, due to poor medical compliance and non-standard early treatment, some patients with joint pain as the primary clinical manifestation are not covered by the diagnostic and therapeutic guidelines, which may lead to delayed treatment. Given the facts above, early diagnosis and treatment of SNRA are equally important as other types of RA patients.

Tenosynovitis is an early manifestation of RA and also a critical cause for functional disability^34^, which may also be a good radiological indicator predicting early development^3,35^. However, tenosynovitis usually presents with joint swelling and pain with poor specificity, which is not easily differentiated from other etiologies and is usually neglected in clinical practice^36^, and there are limited data about its prevalence. Given the facts above, the early diagnosis of active tenosynovitis is particularly important. The diagnosis of this disorder can be helpful to improve new classification criteria, and also prevent the development of damage^9,37,38^.In the present study, the detection rate of affected tendon synovial sheaths upon GS and PD was 39.06% and 30.87% in the SNRA group, respectively, which were higher than 7.71% and 0.94% in the OA group, respectively. Compared with the OA group, total GS scores of tendon synovial sheaths, total PD scores of tendon synovial sheaths, GS scores of tendon synovial sheaths, and average scores were higher in the SNRA group. In the OA group, tenosynovitis with GS grade I was dominant, and there were 68 such affected tendon synovial sheaths (91.89%). No patients positive for tenosynovitis upon PD were detected in the OA group. It was probably because GS-indicated tenosynovitis was not at the active stage in the OA group. The total GS score of tendon synovial sheaths and the total PD score of tendon synovial sheaths were also of a high diagnostic value for SNRA (AUC > 0.85). The finding of tenosynovitis facilitated early diagnosis of SNRA.

Clinically, CRP, ESR and DAS28 are considered indicators for RA activity. DAS28 is an essential indicator of RA activity, but its calculation is more complicated. In addition, ultrasound may still uncover subclinical inflammatory changes of patients in the remission stage of DAS28-defined RA^39^. Synovial inflammation indicates systemic inflammatory response, which is featured by increased serum CRP levels. Therefore, serum CRP levels are proven to be an indicator of synovial inflammation and related to radiological progression^40^. CRP and ESR are the most common laboratory indicators of inflammatory activities, but they are vulnerable to disturbance from other factors, such as infection. In the present study, CRP, ESR and DAS28 in the SNRA group were higher than those in the OA group, indicating that most SNRA patients were at the active stage of inflammation. Meanwhile, except for CRP, which was not correlated with the total BE score in the SNRA group, total GS scores of joints, total PD scores of joints, total BE scores, total GS scores of tendon synovial sheaths, and total PD scores of tendon synovial sheaths were positively correlated with ESR, CRP, and DAS2. The reason may be that BE of joints is a chronic process and may be asynchronous with disease activity. Moreover, serum CRP is prone to be influenced by other factors, such as infection. The correlation between CRP and total BE scores may be further investigated by increasing the sample size. In the SNRA group, ultrasound findings of joints and tendon synovial sheaths were consistent with disease activity, demonstrating that ultrasound is potentially used to assess disease activity.

There are some limitations in the study. First, it was a retrospective single-center study, and the sample size was small, especially the number of OA patients. Second, Of note, the ultrasound scoring systems for joints and tendon synovial sheaths were not designed for OA and did not assess for typical OA features (joint space narrowing, osteophytes). Hence, more informative ultrasound assessment systems should be used in future prospective studies to assess the differences between SNRA and OA patients. Third, all ultrasound evaluations are taken by the same physician, which may be biased and affect the results. Last, only 22 joints and 24 tendon synovial sheaths of the two hands were examined in the two groups. Distal interphalangeal and Carpometacarpal joints those were prone to OA were not included, as the purpose of the study was to use the same US examination protocol for all patients enrolled. What’s more, the diagnostic efficacy of HFUS for joints in other parts of the body remains to be investigated in the future.

In conclusion, HFUS facilitates differential diagnosis of SNRA and OA and the clinical diagnosis of SNRA through assessment of joints and tendon synovial sheaths.

## Data Availability

The datasets generated and/or analysed during the current study are not publicly available, but are available from the corresponding author on reasonable request.
